# Novel *SCN5A* and *GPD1L* Variants Identified in Two Unrelated Han-Chinese Patients With Clinically Suspected Brugada Syndrome

**DOI:** 10.3389/fcvm.2021.758903

**Published:** 2021-12-08

**Authors:** Meng Yuan, Yi Guo, Hong Xia, Hongbo Xu, Hao Deng, Lamei Yuan

**Affiliations:** ^1^Center for Experimental Medicine, The Third Xiangya Hospital, Central South University, Changsha, China; ^2^Department of Medical Information, School of Life Sciences, Central South University, Changsha, China; ^3^Department of Emergency, The Third Xiangya Hospital, Central South University, Changsha, China; ^4^Department of Neurology, The Third Xiangya Hospital, Central South University, Changsha, China; ^5^Disease Genome Research Center, Central South University, Changsha, China

**Keywords:** Brugada syndrome, whole exome sequencing, Han-Chinese patients, minigene splicing assay, novel variants

## Abstract

Brugada syndrome (BrS) is a complexly genetically patterned, rare, malignant, life-threatening arrhythmia disorder. It is autosomal dominant in most cases and characterized by identifiable electrocardiographic patterns, recurrent syncope, nocturnal agonal respiration, and other symptoms, including sudden cardiac death. Over the last 2 decades, a great number of variants have been identified in more than 36 pathogenic or susceptibility genes associated with BrS. The present study used the combined method of whole exome sequencing and Sanger sequencing to identify pathogenic variants in two unrelated Han-Chinese patients with clinically suspected BrS. Minigene splicing assay was used to evaluate the effects of the splicing variant. A novel heterozygous splicing variant c.2437-2A>C in the sodium voltage-gated channel alpha subunit 5 gene (*SCN5A*) and a novel heterozygous missense variant c.161A>T [p.(Asp54Val)] in the glycerol-3-phosphate dehydrogenase 1 like gene (*GPD1L*) were identified in these two patients with BrS-1 and possible BrS-2, respectively. Minigene splicing assay indicated the deletion of 15 and 141 nucleotides in exon 16, resulting in critical amino acid deletions. These findings expand the variant spectrum of *SCN5A* and *GPD1L*, which can be beneficial to genetic counseling and prenatal diagnosis.

## Introduction

Brugada syndrome (BrS), first reported in 1992, is a rare, malignant, life-threatening genetic arrhythmia disorder, having no gross structural abnormality (1–4). It has a complex transmission pattern with incomplete penetrance, involving autosomal dominant inheritance in most cases, and autosomal recessive or X-linked inheritance in a few patients (5, 6). BrS planetwide prevalence is estimated to be 1–16 per 10,000, but the true prevalence is difficult to estimate due to the dynamic electrocardiographic pattern and normal sign during the examination, incomplete penetrance, variable expressivity, and problematic diagnosis (7–10). The characteristic BrS electrocardiogram (ECG) can be classified into three types, referring to various ST-segment and T-wave morphologies in the precordial leads (V1–V3). The classic type 1 pattern shows gradually downsloping coved ST-segment (amplitude ≥2 mm) and negative T-wave in more than one lead (V1–V3). In type 2, there is a saddleback ST-segment with an elevation ≥2 mm and a positive or biphasic T-wave. The type 3 pattern includes a saddleback or coved pattern with ST-segment elevation <1 mm. Out of the three patterns in ECG, types 2 and 3 can convert to type 1 under the induction of sodium channel blockers, and only type 1 is diagnostic for BrS (11–13). Patients with BrS can have unexplained recurrent syncope, migraine, seizures, sleep disturbance, nocturnal agonal respiration, self-terminating polymorphic ventricular tachycardia, ventricular fibrillation (VF), inducibility of ventricular tachycardia with programmed electrical stimulation, and atrial arrhythmia, with a type 1 ECG pattern and sudden cardiac death (SCD) in family members (9, 10, 14, 15). Other cardiac conduction disorders, such as atrioventricular block and right bundle branch block (RBBB) can be accompanied, spontaneously occurred, or induced by drug or other factors (5, 8). A BrS diagnostic score system was proposed in 2016 based on an expert consensus statement on the diagnosis and management of inherited primary arrhythmia syndromes in 2013 and guidelines for ventricular arrhythmia management and SCD prevention in 2015, in which the molecular genetic analysis is included for diagnosis confirmation and as a supplement for clinical tests (13, 16, 17). BrS, which often manifests as syncope, has a comparatively higher prevalence in Southeast Asia than those in Europe or the United States (18). It is eight to ten times more common in adult men than women (19). The mean age of the first episode can be 40 years, while BrS may also occur in infancy or early childhood, even leading to sudden infant death syndrome (15).

A variety of genes and variants are associated with BrS. It can be classified into nine types according to the responsible genes in the Online Mendelian Inheritance in Man (OMIM) database. The most common type appears to be BrS-1 (BRGDA1, OMIM 601144), which accounts for 15–30% of cases and is associated with variants in the sodium voltage-gated channel alpha subunit 5 gene (*SCN5A*, OMIM 600163). Other pathogenic variants were reported in a few patients, and variants in the glycerol-3-phosphate dehydrogenase 1 like gene (*GPD1L*, OMIM 611778) were responsible for <1% of cases, genetically diagnosed as BrS-2 (BRGDA2, OMIM 611777) (5, 8).

In this study, two novel heterozygous variants, c.2437-2A>C in the *SCN5A* gene, and c.161A>T [p.(Asp54Val)] in the *GPD1L* gene, were identified *via* whole exome sequencing (WES) and Sanger sequencing in two unrelated Han-Chinese patients with clinically suspected BrS. This provides significant human data for improving clinical and genetic diagnosis.

## Materials and Methods

### Subjects and Clinical Evaluations

The participants recruited for the present study were two unrelated Han-Chinese patients from Hunan province in southern China who were suspected to have BrS and an unrelated healthy male without related condition and family history as a control. Professional physical examinations, ECG, and blood pressure measurements were performed on patients by two experienced cardiologists. Written informed consent was obtained from subjects before participation. The study complied with the Declaration of Helsinki and was approved by the Institutional Review Board of the Third Xiangya Hospital of Central South University.

### Exome Capture

Genomic DNA was separated from peripheral blood by the phenol-chloroform method (3). WES was performed for screening the pathogenic variants in two patients by BGI-Shenzhen (Shenzhen, China). One microgram genomic DNA was randomly fragmented using the Covaris technique in which 150–250 bp fragments were selected. DNA fragments were subjected to end-repairing, A-tailing reactions, and adaptor ligation, which were further used for amplification and sequencing. They were further purified and hybridized to the BGI exon array. The captured qualified circular DNA library by rolling circle amplification was sequenced on the BGISEQ-500 sequencing platform (20, 21).

### Variants Calling and Validation

The clean reads obtained by trimming and filtering raw reads were aligned with the human reference genome (GRCh37/hg19) by Burrows-Wheeler Aligner (BWA, v0.7.15) (22). Picard (v2.5.0, http://broadinstitute.github.io/picard/) removed duplicate reads. Genome Analysis Toolkit (GATK, v3.3.0, http://www.broadinstitute.org/gatk/guide/best-practices) tools performed local realignment and base quality score recalibration. HaplotypeCaller in GATK obtained credible single nucleotide polymorphisms (SNPs) and insertions/deletions (InDels) (23). SnpEff software (http://snpeff.sourceforge.net/SnpEff_manual.html) was used for variant annotation and prediction (24). The following databases were used to filter candidate variants, including the Human Gene Mutation Database (HGMD), the Single Nucleotide Polymorphism database (version 154, dbSNP154), ClinVar, National Heart, Lung and Blood Institute Exome Sequencing Project (ESP6500), Genome Aggregation Database (gnomAD), 1000 Genomes Project (1000G), the China Metabolic Analytics Project (ChinaMAP) database and the in-house BGI exome database (BGI-Shenzhen, Shenzhen, China) with 1,943 Han-Chinese controls (21, 25, 26). Variant pathogenicity was predicted by MutationTaster, Protein Variation Effect Analyzer (PROVEAN), Sorting Intolerant from Tolerant (SIFT), MutationAssessor, Combined Annotation Dependent Depletion (CADD, v1.4), the Berkeley Drosophilia Genome Project (BDGP) splice site prediction tool (v0.9), and NetGene2 server (21, 27). Sanger sequencing was performed on the ABI3500 sequencer (Applied Biosystems Inc., Foster City, CA, U.S.A.) for the patients to verify the candidate variants as previously described (28). The following were the paired primer sequences for validating variants in Patient 1 and Patient 2, respectively: 5′-GCTTTCAGGCAGGAGCTAGA-3′ and 5′-TGATGGCTAGCACCAGTGTC-3′, 5′-CCGCCCAAGTGAGTTTATGT-3′, and 5′-TCACCCCAAAGTCTTACCACA-3′. The detected variants were further categorized according to the American College of Medical Genetics and Genomics (ACMG) guidelines for the sequence variant interpretation (27).

Basic Local Alignment Search Tool (BLAST, https://blast.ncbi.nlm.nih.gov/Blast.cgi) was used for analyzing protein sequences in nine different species arranged in evolutionary order. The SWISS-MODEL tool (http://www.swissmodel.expasy.org) and PyMOL software (version 2.3, Schrödinger, LLC, Portland, U.S.A.) predicted and visualized wildtype and variant protein structures, respectively (29).

### Minigene Splicing Assay

The minigene splicing assay was performed for the splicing variant. The wildtype (WT) and mutant (MT) forms of the minigene constructs composed of two parts, encompassing *SCN5A* exons 15–17, intron 15, and partial intron 16, were amplified from genomic DNA of Patient 1, using the following primer pairs: 5′-AAGCTTGGTACCGAGCTCGGATCCGTCTTCACAGGGATTTTCACAGCAGAGA-3′ and 5′-CTGTAGGGCATTGGGTGAGTGGACAGATGGTTGATGGA-3′, 5′-ACTCACCCAATGCCCTACAGCAGCAGCCCCAGGCCTCT-3′, and 5′-TTAAACGGGCCCTCTAGACTCGAGCTGCTTGCTGGACTCCTCCTCCGTGCCC-3′, and the amplified products were then introduced into the pMini-CopGFP vector (Beijing Hitrobio Biotechnology Co., Ltd., Beijing, China). The vector was double digested at BamHI and XhoI sites. The cloning was achieved by using ClonExpress II One Step Cloning Kit (Vazyme, Nanjing, China). The WT and MT forms of minigene constructs were verified by Sanger sequencing and selected for transfection. Human embryonic kidney (HEK) 293T cells were prepared in Dulbecco's modified Eagle's medium supplemented with 10% fetal bovine serum (HyClone, Logan, Utah, U.S.A.) at 37°C and 5% CO_2_. Transfection of minigene constructs was performed using the Lipofectamine 2000 reagent (Invitrogen, Carlsbad, CA, U.S.A.), in accordance with the instructions of the manufacturer. At 48-h post-transfection, cells were collected for RNA extraction by using Trizol reagent (Cowin Biotech Co., Ltd., Taizhou, China). Reverse transcription-polymerase chain reaction (RT-PCR) was performed with primers: 5′-GGCTAACTAGAGAACCCACTGCTTA-3′ and 5′-GTTTAAACGGGCCCTCTAGACTCGA-3′, and the products were separated by electrophoresis analysis. Sanger sequencing was applied to identify the isoforms.

## Results

### Clinical Features

**Figure 1:**
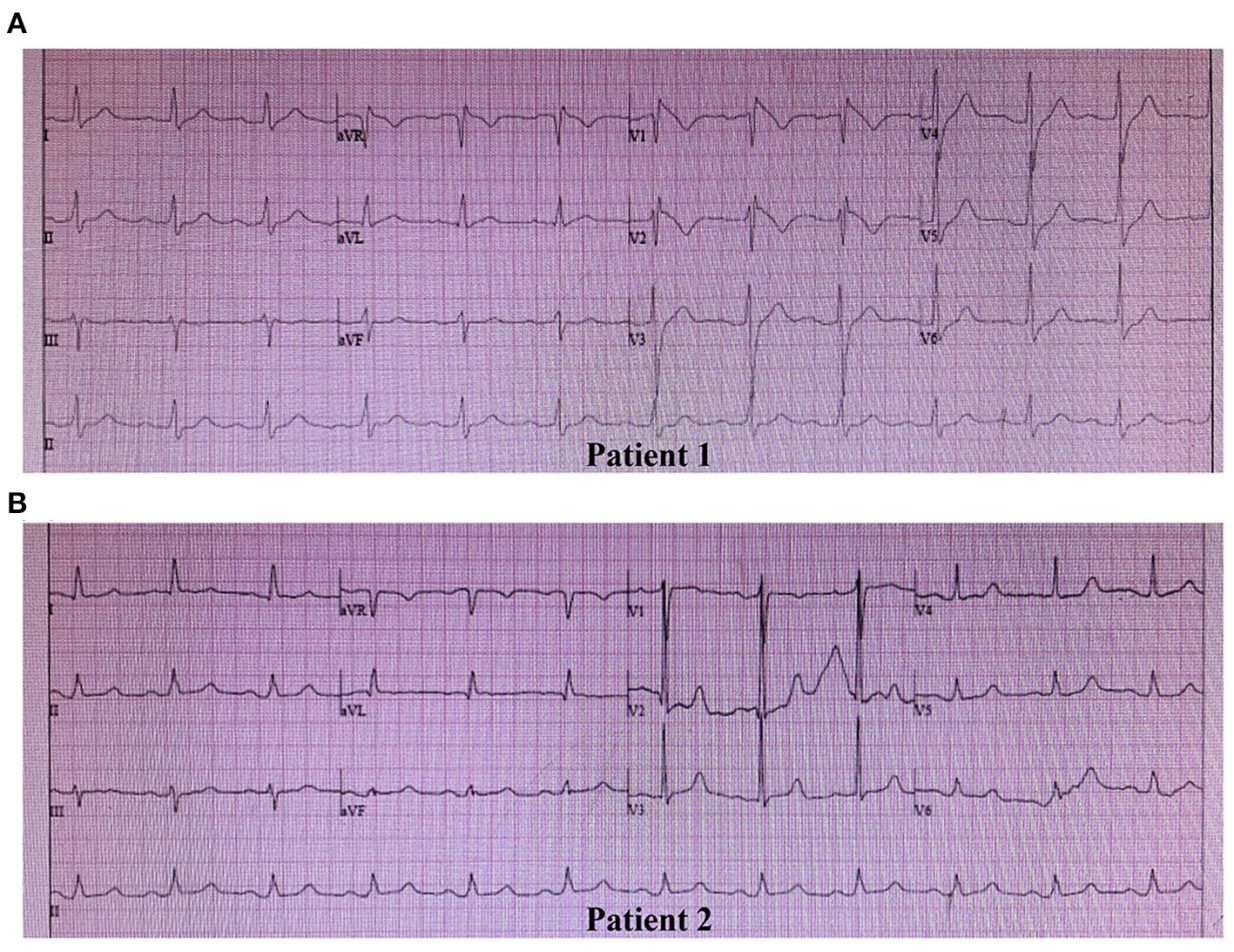
Spontaneous electrocardiogram (ECG) of two patients. **(A)** ECG of Patient 1: Type 1 Brugada pattern displaying a coved-type ST-segment (≥2 mm) and negative T wave in V1 and V2, a first-degree atrioventricular block pattern, and an incomplete right bundle branch block. **(B)** ECG of Patient 2: Type 3 Brugada pattern displaying a saddleback ST-segment (<1 mm) in V2, and a first-degree atrioventricular block pattern.

Clinical characteristics and ECG findings of patients are summarized in Table 1. Patient 1, a 42-year-old male, was admitted to the hospital suffering from recurrent syncope (eight times in 8 h), and VF in ECG was detected by the rescue team. ECG suggested a type 1 Brugada pattern, accompanied by a first-degree atrioventricular block and an incomplete RBBB (Figure 1A). He had high systolic blood pressure (157/83 mmHg). The condition in Patient 1 was in line with the diagnostic criteria of BRGDA1.

**Table 1:** Clinical characteristics of two patients.

**Case**	**Patient 1**	**Patient 2**
Sex	Male	Male
Age (years)	42	81
Recurrent syncope	+	–
Nocturnal agonal respiration	–	+
Dizziness	–	+
Palpitation	–	+
Angina	–	+
Migraine	–	–
Seizure	–	–
Sleep disturbance	–	+
Sudden cardiac death in family member (<45 years)	-	+
Brugada electrocardiogram pattern	Type 1	Type 3
Ventricular fibrillation	+	–
Atrioventricular block	First degree	First degree
Right bundle branch block	Incomplete	–

Patient 2 was an 81-year-old male, whose father died of SCD at the age of 44 years. The patient had post-hepatitis cirrhosis and was admitted to the hospital due to gastroesophageal variceal bleeding. His medical history revealed coronary heart disease, grade 3 hypertension (180/100 mmHg), brain atrophy, right renal cysts, chronic cholecystitis, chronic bronchitis, hypoproteinemia, and anemia. He complained of nocturnal agonal respiration, dizziness, palpitation, angina, and sleep disturbance. ECG suggested a type 3 Brugada pattern accompanied by a first-degree atrioventricular block (Figure 1B). He was in poor physical shape and passed away shortly after discharge with a highly suspected diagnosis of BrS.

### Variant Screening and Bioinformation Analysis

**Figure 2:**
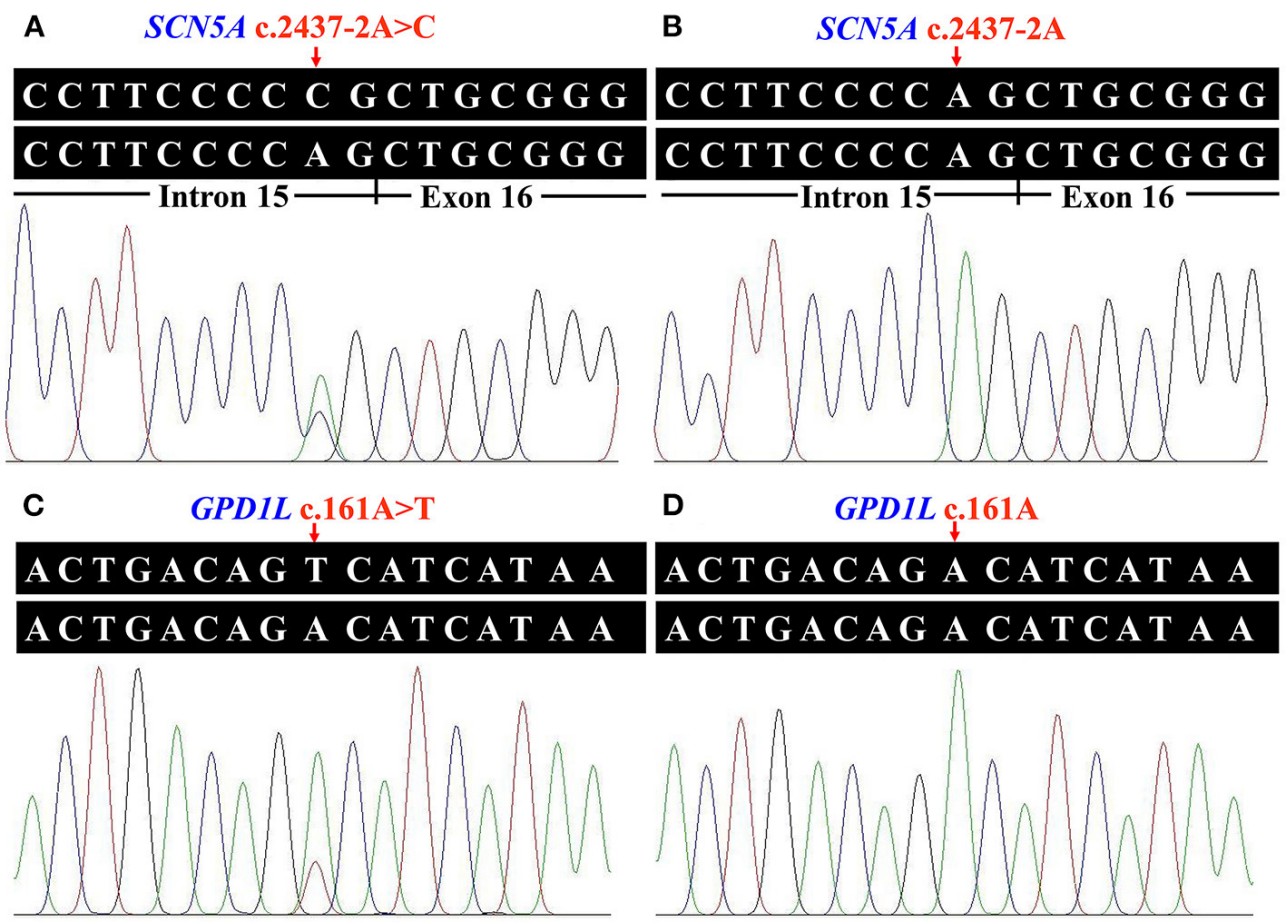
Identified variants of the sodium voltage-gated channel alpha subunit 5 gene (*SCN5A*) and the glycerol-3-phosphate dehydrogenase 1 like gene (*GPD1L*) in the two patients. **(A)** Heterozygous transversion (c.2437-2A>C) at the splice acceptor site of *SCN5A* intron 15 in Patient 1. **(B)** The normal *SCN5A* sequence of an unaffected individual. **(C)** The *GPD1L* sequence with heterozygous c.161A>T [p.(Asp54Val)] variant in Patient 2. **(D)** The normal *GPD1L* sequence of an unaffected individual.

Whole exome sequencing performed on the genomic DNA of Patient 1 produced 115.33 million total effective reads with 99.90% mapped to the human reference sequence, while a total of 153.24 million total effective reads with 99.86% mapped to the human reference sequence were generated with the genomic DNA of Patient 2. For all analyzed genes, the average sequencing depth of the target region was 144.82× for Patient 1 and 192.93× for Patient 2. Of these sequences, 99.24% and 99.57% of the target regions were covered by at least 10×. About 91,330 SNPs and 14,265 InDels were detected in Patient 1. There were 97,957 SNPs and 16,106 InDels detected in Patient 2. These variants were filtered by HGMD, dbSNP154, ClinVar, ESP6500, gnomAD, 1000G, ChinaMAP database, and in-house BGI exome database to assess possible pathogenic variants in the patients. MutationTaster, PROVEAN, SIFT, MutationAssessor, CADD, BDGP splice site prediction tool, and NetGene2 server predicted the potential variants to be deleterious (Table 2). After screening the above databases and analyzing all known BrS-associated genes, only two novel and damaging heterozygous variants, c.2437-2A>C in the *SCN5A* gene (NG_008934.1, NM_001099404.1) and c.161A>T [p.(Asp54Val)] in the *GPD1L* gene (NM_015141.3, NP_055956.1), were considered as potential disease-causing variants in Patient 1 and Patient 2, respectively. Sanger sequencing confirmed these variants (Figure 2). The c.2437-2A>C variant was classified as “pathogenic” (PVS1 + PM2 + PP3), and the c.161A>T variant was classified as “likely pathogenic” (PM1 + PM2 + PP2 + PP3), following the ACMG guidelines.

**Table 2:** Identification of variants in the patients.

**Variant**	**Variant 1**	**Variant 2**
Gene	*SCN5A*	*GPD1L*
Exon	/	2
Intron	15	/
Nucleotide change	c.2437-2A>C	c.161A>T
Amino acid change	p.(Arg814_Leu818del)/ p.(Arg814_Leu860del)	p.(Asp54Val)
Zygosity	Heterozygote	Heterozygote
Variant type	Splicing	Missense
HGMD	No	No
dbSNP154	No	No
ClinVar	No	No
ESP6500	No	No
gnomAD	No	No
1000G	No	No
ChinaMAP database	No	No
In-house BGI exome database	No	No
MutationTaster (probability, prediction)	/	~1, disease causing
PROVEAN (score, prediction)	/	−4.44, deleterious
SIFT (score, prediction)	/	0.033, damaging
MutationAssessor (score, functional impact)	/	2.76, medium
CADD v1.4 (phred-score, prediction)	/	26.6, deleterious
BDGP v0.9	Destroy the acceptor site	/
NetGene2 server	Destroy the acceptor site	/

*HGMD, Human Gene Mutation Database; dbSNP154, Single Nucleotide Polymorphism database (version 154); ESP6500, Exome Sequencing Project 6500; gnomAD, Genome Aggregation Database; 1000G, 1000 Genomes Project; ChinaMAP, China Metabolic Analytics Project; PROVEAN, Protein Variation Effect Analyzer; SIFT, Sorting Intolerant from Tolerant; CADD, Combined Annotation Dependent Depletion; BDGP, Berkeley Drosophila Genome Project*.

**Figure 3:**
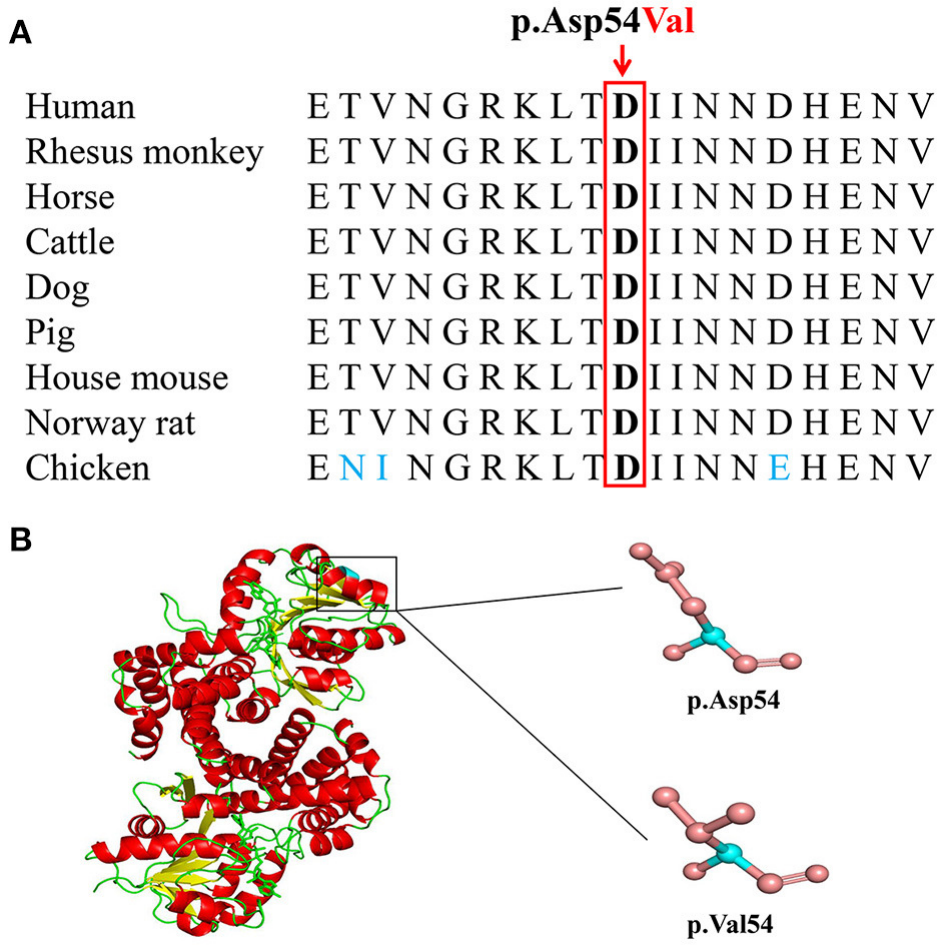
The conserved analysis and structural prediction of wildtype and mutant glycerol-3-phosphate dehydrogenase 1 like (GPD1L) proteins. **(A)** The GPD1L p.Asp54 amino acid residue is conserved in nine species by a multiple sequence alignment program. **(B)** Structural prediction of wildtype and variant GPD1L proteins.

Basic Local Alignment Search Tool comparison of protein sequences from human to chicken revealed that p.Asp54 was highly conserved in GPD1L protein (Figure 3A). A structural model showed the conformational alteration of aspartic acid (Asp-54) into valine (Val-54), further confirming the possible pathogenicity of the variant (Figure 3B).

### Splicing Analysis of *SCN5A* c.2437-2A>C in the Minigene

**Figure 4:**
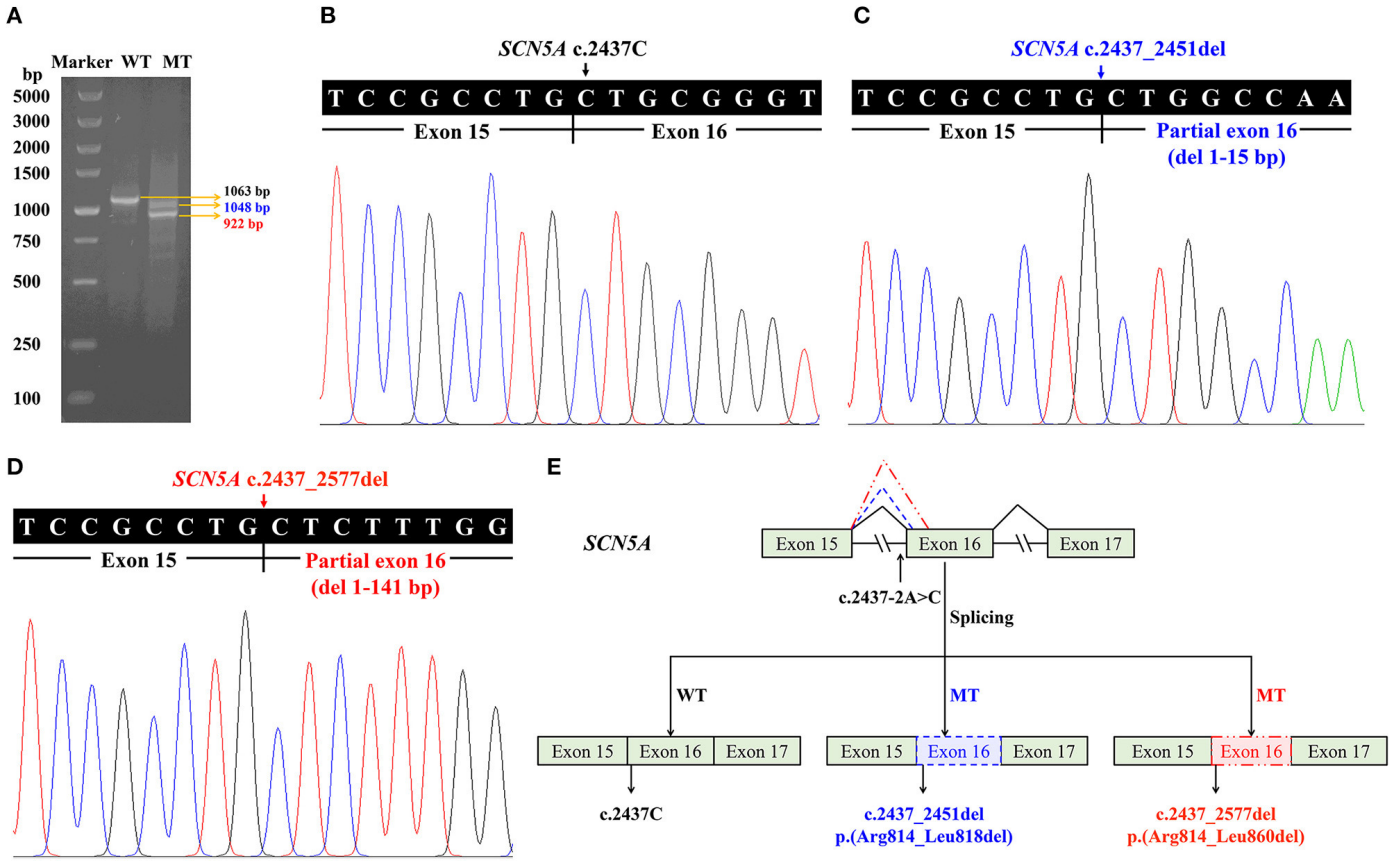
Minigene assay for the sodium voltage-gated channel alpha subunit 5 gene (*SCN5A*) c.2437-2A>C variant and the schematic splicing model. **(A)** Electrophoresis of reverse transcription PCR products, with mRNA extracted from cells transfected with pMini-CopGFP-*SCN5A* (WT for wildtype: 1,063 bp, MT for mutant: 1,048/922 bp). **(B)** Sanger sequencing of the wildtype (1,063 bp) shows a junction between exons 15 and 16. **(C)** Sanger sequencing of the mutant (1,048 bp) indicates a deletion of 15 bp from exon 16. **(D)** Sanger sequencing of the mutant (922 bp) indicates a deletion of 141 bp from exon 16. **(E)** A schematic splicing mechanism of the *SCN5A* c.2437-2A>C variant. The continuous lines between exons show the normal splicing of the wildtype, and the dotted lines demonstrate the aberrant splicing, resulting in activation of two cryptic sites and partial loss of exon 16.

Minigene assay and RT-PCR analysis were performed to identify the abnormal splicing. Electrophoresis analysis of RT-PCR products showed a single band for WT and two shorter bands for MT (Figure 4A). Sanger sequencing indicated normal splicing in exons 15 and 16 for WT (Figure 4B), and partial loss of exon 16 for MT, including the deletion of the first 15 nucleotides (c.2437_2451del) and 141 nucleotides from exon 16 (c.2437_2577del) (Figures 4C,D). It indicated that the *SCN5A* c.2437-2A>C variant can abolish the intron 15 canonical acceptor splice site and lead to activation of two cryptic sites in exon 16, predicted to cause in-frame deletions, p.(Arg814_Leu818del) and p.(Arg814_Leu860del) (Figure 4E).

## Discussion

**Figure 5:**
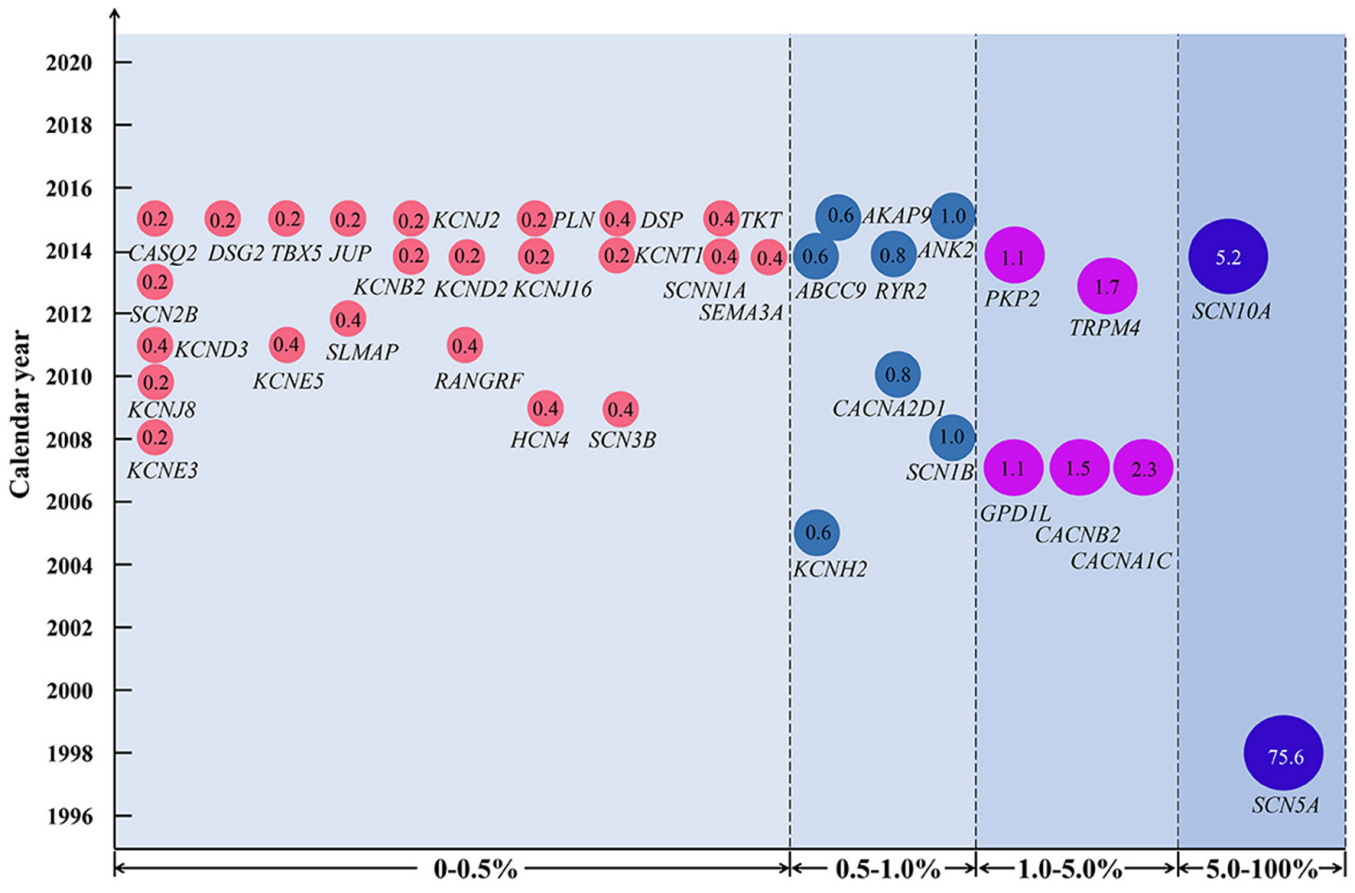
Variant frequency of genes related to Brugada syndrome (BrS). Each disease-causing or susceptibility gene corresponds to the year when it was first discovered to be related to BrS. The number in the circle indicates the percentage of variant frequency. The gene symbol used is approved by the HUGO Gene Nomenclature Committee.

Brugada syndrome seems to have a poor genotype-phenotype correlation, with obvious genetic and phenotypic heterogeneity (30, 31). Incomplete penetrance and even asymptomatic gene carriers have been commonly reported (30, 32). Since *SCN5A* was reported as a causative gene of BrS in 1998, a great number of variants have been identified in more than 36 pathogenic or susceptibility genes associated with BrS in the past 2 decades (Figure 5) (33–35).

**Figure 6:**
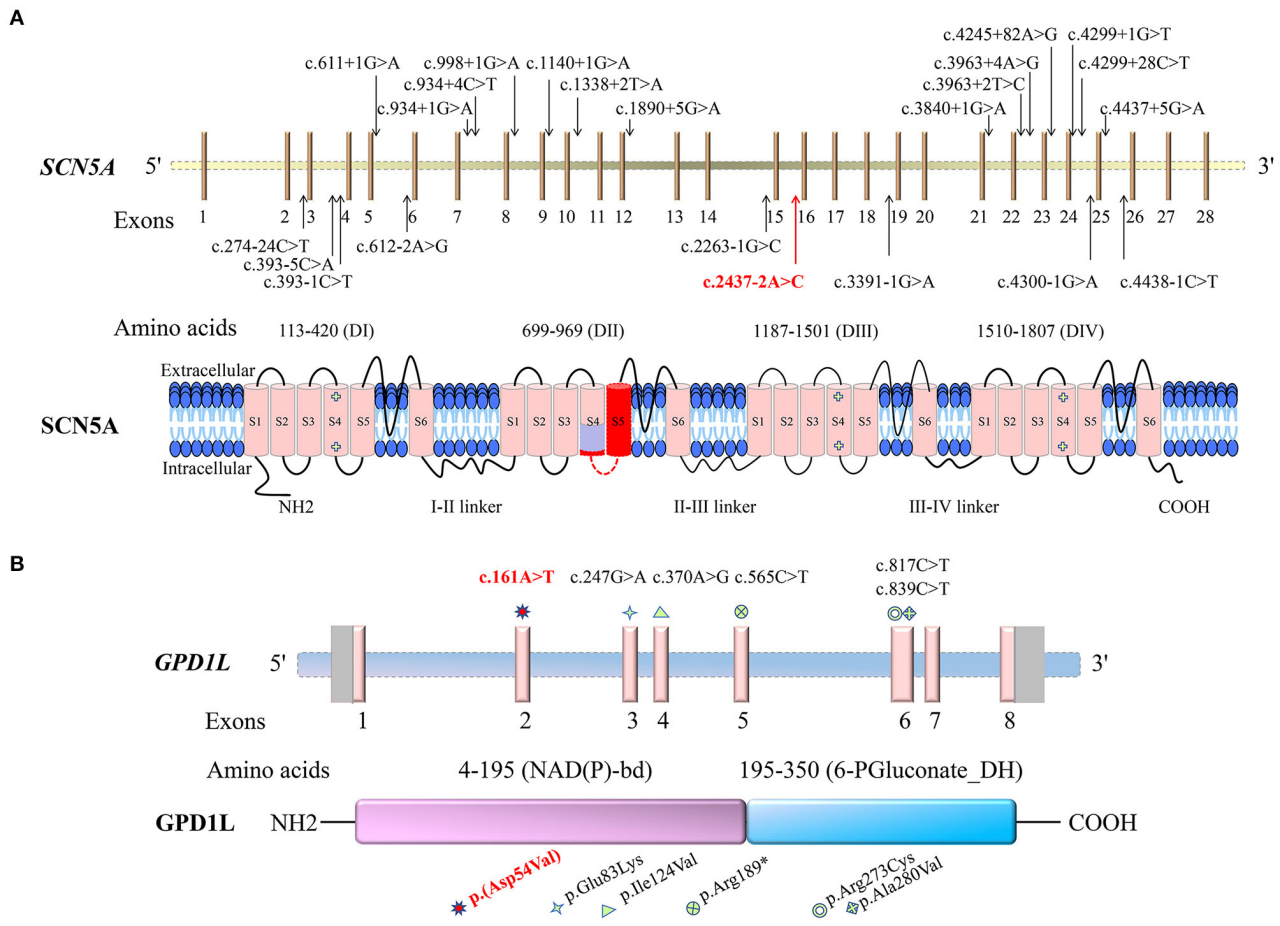
Schematic representations of the sodium voltage-gated channel alpha subunit 5 gene (*SCN5A*) and the glycerol-3-phosphate dehydrogenase 1 like gene (*GPD1L*) and the encoded proteins, as well as the identified variants. Variants identified in this study are indicated by red colors. **(A)** The Brugada syndrome-1-related single nucleotide substitutions affecting splicing are shown in the *SCN5A* gene. SCN5A consists of four putative transmembrane domains (DI-DIV), with six transmembrane segments (S1–S6) for each section. The light purple-colored region on SCN5A is the segment encoded by the first 15 nucleotides of exon 16, and the dark red is encoded by the 16–141 nucleotides of exon 16. **(B)** The *GPD1L* gene variants related to Brugada syndrome-2 and sudden infant death syndrome are shown. GPD1L contains the glycerol-3-phosphate dehydrogenase (GPD) motif [nicotinamide adenine dinucleotide (phosphate)-binding domain, NAD(P)-bd] and 6-phosphogluconate dehydrogenase C-terminal domain-like (6-PGluconate_DH).

In the present study, c.2437-2A>C in the *SCN5A* gene was prosecuted as the pathogenic variant for Patient 1 with BRGDA1, and c.161A>T [p.(Asp54Val)] in the *GPD1L* gene was considered as the potential pathogenic variant in Patient 2 with possible BRGDA2 lacking an available drug challenge test. Currently, more than 950 *SCN5A* gene variants have been reported, and more than 360 variants have been recorded as responsible for BRGDA1 (http://www.hgmd.cf.ac.uk/ac/index.php, http://www.lovd.nl/3.0/home). The single nucleotide substitution affecting splicing accounted for 6% of BRGDA1 (Figure 6A). However, only three substitutions in the *GPD1L* gene, c.370A>G (p.Ile124Val), c.565C>T (p.Arg189^*^), and c.839C>T (p.Ala280Val), have been found to be related to BRGDA2 (Figure 6B) (36). The c.2437-2A>C variant was located at the splice acceptor site of intron 15 in the *SCN5A* gene, and this transversion was predicted to destroy the splice acceptor site by the BDGP splice site prediction tool and NetGene2 server. *In silico* programs (MutationTaster, PROVEAN, SIFT, MutationAssessor, and CADD) predicted that the c.161A>T transversion in the *GPD1L* gene would be deleterious. The conclusion that these variants were pathogenic is further supported by their absence in public variant databases and the 1,943 Han-Chinese controls of the in-house BGI exome database.

Sodium voltage-gated channel alpha subunit 5 gene, located at chromosome 3p22.2, has 28 exons and encodes the α-subunit of the cardiac voltage-gated sodium channel (Na_v_1.5) protein, which contains 2016-amino acid (15, 37, 38). The highly cardiac-specific Na_v_1.5 is located in T-tubule membranes and intercalated discs in cardiomyocytes (http://biogps.org) (39, 40). It consists of four putative homologous repetitive transmembrane domains: DI (113–420 amino acids), DII (699–969 amino acids), DIII (1,187–1,501 amino acids), and DIV (1,510–1,807 amino acids). Each domain contains six transmembrane segments (S1-S6), including five hydrophobic regions (S1–S3, S5, and S6) and a positively charged segment (S4), probably acting as a voltage sensor (https://www.uniprot.org/uniprot/, Figure 6A) (33). The Na_v_1.5 channel is able to conduct a depolarized sodium inward current, forming a rapidly rising action potential in an activated process, which plays an important role in cardiomyocyte excitability and normal electrical pulse conduction (38, 41).

The c.2437-2A>C variant in the *SCN5A* gene is located in the 3' splice junctions at the boundary between intron 15 and exon 16, where canonical AG dinucleotides appear. Minigene assay showed that the variant destroyed the canonical acceptor site and activated the cryptic splice sites in exon 16, predicted to cause in-frame deletion of 5 [p.(Arg814_Leu818del)] and 47 amino acids [p.(Arg814_Leu860del)], corresponding to the transmembrane segments of S4 and S5 in DII of Na_v_1.5 protein, which is critical to the voltage-sensor function. Previous studies showed that BrS-related *SCN5A* variants could lead to loss of function of the sodium channel mediated by haploinsufficiency (42, 43), or the related variant channels exert a dominant-negative effect on the WT channels (41). The mechanism of BrS caused by the c.2437-2A>C variant in Patient 1 may be similar to the pathogenesis of variants in nearby residues of the same domain, which is related to loss of function of the sodium channel (44).

Heterozygous *SCN5A*-p.Arg367His patient-specific-induced pluripotent stem cell-derived cardiomyocytes had decreased sodium inward current density, changed voltage-dependent curves in activation and inactivation, and accelerated recovery from inactivation (45). Transgenic zebrafish with the cardiac expression of human *SCN5A* p.Asp1275Asn variant revealed some clinical manifestations related to BrS, such as conduction disorders and early death (46). *Scn*5*a*^−/−^ mice had severe defects to embryonic lethality, while *Scn*5*a*^+/−^ mice were viable and showed decreased sodium channel density and slowed conduction (47).

Glycerol-3-phosphate dehydrogenase 1 like gene, located at chromosome 3p22.3, a position near to *SCN5A*, includes eight exons and expresses a 351-amino acid membrane-associated protein with a molecular mass of 40 kD. The protein is highly expressed in the heart (34, 48). It contains N-terminal glycerol-3-phosphate dehydrogenase (GPD) motif (nicotinamide adenine dinucleotide (phosphate)-binding domain, 4–195 amino acids) and 6-phosphogluconate dehydrogenase C-terminal domain-like (195–350 amino acids), in which amino acids 22–28 are highly homologous to amino acids 830–836 in the transmembrane domain of Na_v_1.5 (Figure 6B) (34, 36). As a member of the GPD family, GPD1L has an 84% amino acid homology to GPD1 and is involved in glucose and nicotinamide adenine dinucleotide-dependent energy metabolism, mammalian respiratory chain, and glycerophosphate shuttle (49, 50). It may have a cardiac-specific physiological function and may be coupled with Na_v_1.5, which can link the myocardial metabolic state to cellular excitability by modulating sodium current density, responsible for cardiac conduction disorder (48).

The c.161A>T variant in the *GPD1L* gene of exon 2 may lead to an aspartic acid-to-valine substitution, p.(Asp54Val), changing from a negatively charged acidic hydrophilic amino acid residue to a neutral hydrophobic amino acid residue, which may impact the tertiary structure or function. Aspartic acid at position 54 was conserved in different species by a multiple sequence alignment program, supporting that the amino acid change may lead to protein function changes. The variant p.(Asp54Val), located in the nicotinamide adenine dinucleotide (phosphate)-binding domain, may have the same pathogenic mechanism as the sudden infant death syndrome-related p.Glu83Lys variant in the same domain and the BrS-related p.Ala280Val variant outside the domain. Both variants have been shown to result in the decrease of enzyme activity and the increase of substrate glycerol-3-phosphate, which further leads to increased Na_v_1.5 phosphorylation *via* the GPD1L-dependent pathway, significantly decreasing the sodium current density and leading to the conduction disorder (48).

## Conclusions

A novel splicing variant c.2437-2A>C in the *SCN5A* gene and a novel missense variant c.161A>T [p.(Asp54Val)] in the *GPD1L* gene were identified in two unrelated Han-Chinese patients with BRGDA1 and possible BRGDA2, respectively. These findings expand the variant spectrum of *SCN5A* and *GPD1L*, which can be beneficial to genetic counseling and prenatal diagnosis. Universal, affordable, and efficient WES has the potential to uncover unsuspected rare or common variants for heterogeneous disorders, like BrS (51–53). The combined strategy of WES and Sanger sequencing can facilitate timely diagnoses and optimal care for those with clinically suspected BrS, presenting weak disease evidence. The limitation of this study is that the influence of oligogenic inheritance, background genotype, and environmental factors on BrS cannot be excluded. Due to the privacy concerns of Patient 1 and the death of Patient 2 without an offspring, co-segregation analysis was limited, and identification of the same variants in more confirmed patients will help to further support the pathogenicity of c.2437-2A>C in the *SCN5A* gene and determine the pathogenicity of c.161A>T in the *GPD1L* gene. Further functional studies *in vitro* and/or *in vivo* will help to elucidate the potential pathogenic mechanism of BrS.

## Data Availability Statement

According to national legislation/guidelines, specifically the Administrative Regulations of the People's Republic of China on Human Genetic Resources (http://www.gov.cn/zhengce/content/2019-06/10/content_5398829.htm, http://english.www.gov.cn/policies/latest_releases/2019/06/10/content_281476708945462.htm), no additional raw data is available at this time. Data of this project can be accessed after an approval application to the China National GeneBank (CNGB, https://db.cngb.org/cnsa/). Please refer to https://db.cngb.org/, or email: CNGBdb@cngb.org for detailed application guidance. The accession code CNP0002245 should be included in the application.

## Ethics Statement

The studies involving human participants were reviewed and approved by the Institutional Review Board of the Third Xiangya Hospital of Central South University. The patients/participants provided their written informed consent to participate in this study. Written informed consent was obtained from the participants or their legal guardian/next of kin for the publication of any potentially identifiable images or data included in this article.

## Author Contributions

MY, YG, and LY conceived and designed this study. YG, HXia, and LY collected the patient samples and clinical data. MY, YG, and HXu performed the experiments. MY, HD, and LY analyzed the data. MY, YG, HD, and LY wrote the manuscript. The final version of the manuscript was read and approved by all authors.

## Funding

This study was supported by the National Natural Science Foundation of China (Grant Nos. 81800219 and 81873686), Natural Science Foundation of Hunan Province (Grant Nos. 2019JJ50927, 2020JJ3057, and 2020JJ4830), Scientific Research Project of Health Commission of Hunan Province, China (Grant No. B2019174), the Wisdom Accumulation and Talent Cultivation Project of the Third Xiangya Hospital of Central South University (Grant No. YX202109), and Distinguished Professor of the Lotus Scholars Award Program of Hunan Province, China.

## Conflict of Interest

The authors declare that the research was conducted in the absence of any commercial or financial relationships that could be construed as a potential conflict of interest.

## Publisher's Note

All claims expressed in this article are solely those of the authors and do not necessarily represent those of their affiliated organizations, or those of the publisher, the editors and the reviewers. Any product that may be evaluated in this article, or claim that may be made by its manufacturer, is not guaranteed or endorsed by the publisher.
